# Supplementary figures and images for: Dynamics of postnatal bone development and epiphyseal synostosis in the caprine autopod
Source: Dev Dyn. 2025 May 13;255(4):417–29. doi: 10.1002/dvdy.70038 (PMC12353835; doi:10.1002/dvdy.70038)

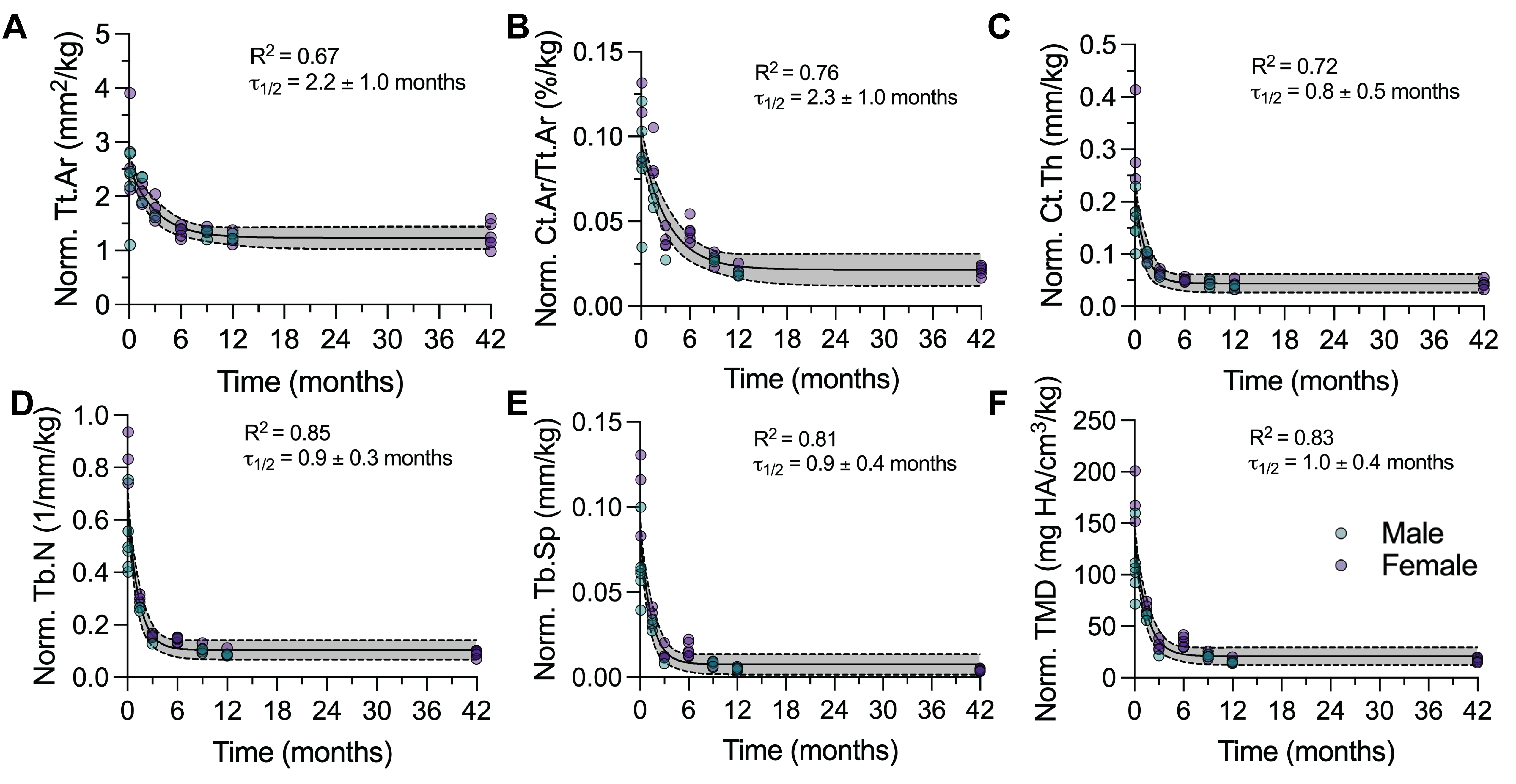

Supplement: Supplementary file 1 — FIGURE S1. Morphodynamic changes of P1 cross‐sectional distribution correlate with ambulatory loads. Body mass normalized (A) Cortical tissue cross‐sectional area (Tt.Ar), (B) Cortical area fraction (Ct.Ar/Tt.Ar), (C) Cortical thickness (Ct.Th), (D) Trabecular number (Tb.N), (E) Trabecular spacing (Tb.Sp), and (F) Trabecular tissue mineral density (TMD). Prefix “Norm” indicates outcome is normalized to animal mass. Data graphed as individual data points versus time fit with a one‐phase decay model. Gray bands indicate 95% confidence interval for each curve for the full dataset. Datapoint color indicates animal sex: male = teal, female = purple. Goodness of fit (R 2) and half‐life (τ 1/2) are reported for each graph. [file DVDY-255-417-s001.tif]
